# Supplementary material for: Chemical Solution Deposition of Ordered 2D Arrays of Room-Temperature Ferrimagnetic Cobalt Ferrite Nanodots
Source: Polymers (Basel). 2019 Sep 30;11(10):1598. doi: 10.3390/polym11101598 (PMC6835257; doi:10.3390/polym11101598)
Supplement: Supplementary file 1 [file polymers-11-01598-s001.pdf]

Supplementary Material

Chemical Solution Deposition of Ordered Arrays of Room-Temperature Ferrimagnetic Cobalt Ferrite Nanodots

Jin Xu, Justin Varghese, Giuseppe Portale, Alessandro Longo, Jamo Momand, Ali Syari'ati, Jeroen A. Heuver, Petra Rudolf, Bart J. Kooi, Beatriz Noheda, Katja Loos

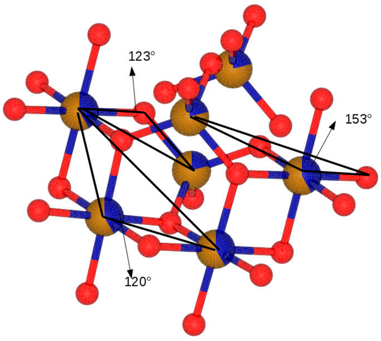

Figure S1. Schematic representation of the spinel structure, where the red spheres represent O atoms, the blue/yellow spheres represent the Co or Fe atoms, and the blue/red sticks represent the atomic bonds. The black lines are the visual aids marking the three Co–O···M or O–Co···M alignments considered in the three-body simulation.

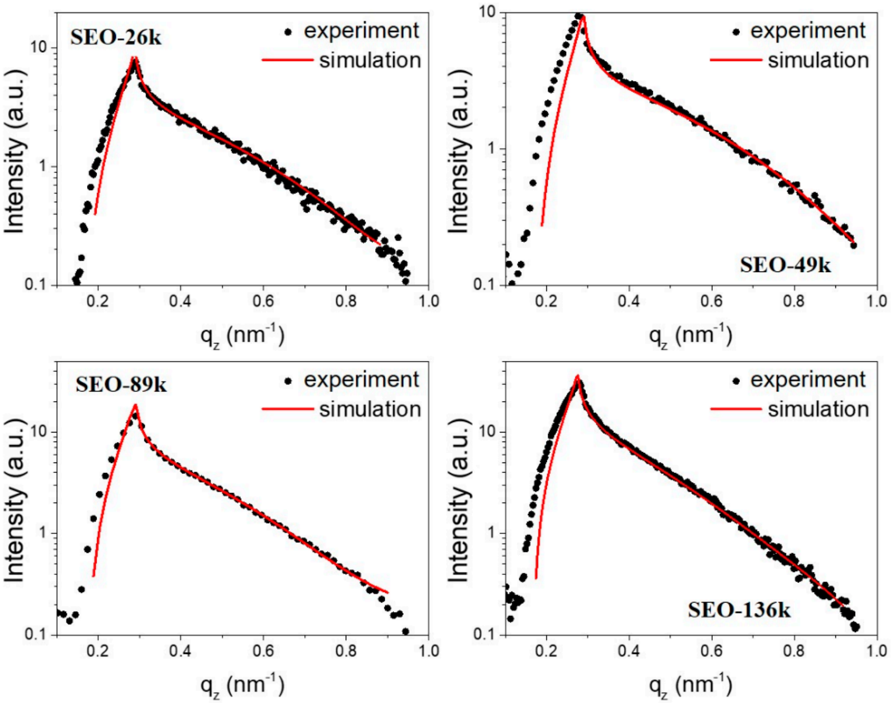

Figure S2. 1D GISAXS plots along the q_z direction of the CFO nanodots prepared from different templates, in which the red curves are the simulation results, and the black points are the experimental results.

Table S1. Structural parameters obtained from the simulated and experimental GISAXS results, where q_1 is the position of the first scattering peak along q_y and inter-row distance is the distance between two rows of dots.

Template	SEO-26k	SEO-49k	SEO-89k	SEO-136k
q_1 (nm^{-1})	0.22	0.20	0.12	0.09
Inter-row Distance (nm)	29.0	30.8 ± 4.0	46.5 ± 9.7	53.8 ± 26.9
Lattice type	Paracrystalline HEX 2D	Paracrystalline HEX 2D	Paracrystalline 1D	Paracrystalline 1D
Diameter (nm)	20.1	20 ± 2.6	26.3 ± 5.5	30 ± 15
Height (nm)	7.3	6.7 ± 0.9	8.6 ± 1.8	9.0 ± 4.5

Table S2. Two-body simulation results at Fe K-edge of the CFO standard and the nanodots annealed at 950 °C, where CN is the coordination number, R is the atomic distance, and σ is the Debye–Waller factor indicating the static and thermal disorder of the shell.

Shell	CFO nanodots			CFO commercial nanopowder		
	**CN**	**R(Å)**	σ^2 (Å^2)	**CN**	**R(Å)**	σ^2 (Å^2)
Fe^1–O	4.0	1.86	0.003	3.6	1.84	0.002
Fe^2–O	5.1	2.06	0.001	5.4	2.07	0.003
$Fe^2 \cdots M^2$	5.1	3.00	0.003	5.4	3.00	0.007
$Fe^2 \cdots M^1$	5.1	3.50	0.002	5.4	3.52	0.010
$Fe^1 \cdots M^2$	11.9	3.52	0.003	10.8	3.46	0.001

Table S3. Three-body simulation results at Fe K-edge of the CFO standard and the nanodots annealed at 950 °C.

Atoms	O–Fe^1–O	O–$Fe^1 \cdots M^2$	O–$Fe^2 \cdots M^1$	$M^2 \cdots Fe^2 \cdots M^2$
Nanopowder θ (°)	120	85.47	153.70	120.70
Nanodots θ (°)	120	80.44	153.70	120.70

Table S4. Magnetic parameters of the nanodots, in which H_c is the coercive field of the nanodots at 300 K.

Template	Dot Diameter (nm)	Dot Height (nm)	H_c (Oe)	T_b (K)
SEO-26k	20.1	7.3	89	312
SEO-49k	20 ± 2.6	6.7	96	323
SEO-89k	26.3 ± 5.5	8.6	282	343
SEO-136k	30 ± 15	9	366	350
